# Distributed Leader-Following Finite-Time Consensus Control for Linear Multiagent Systems under Switching Topology

**DOI:** 10.1155/2014/248041

**Published:** 2014-04-24

**Authors:** Xiaole Xu, Shengyong Chen, Lixin Gao

**Affiliations:** ^1^Wenzhou Vocational College of Science & Technology, Zhejiang 325006, China; ^2^College of Information Engineering, Zhejiang University of Technology, Zhejiang 310023, China; ^3^Institute of Intelligent Systems and Decision, Wenzhou University, Zhejiang 325027, China

## Abstract

This paper investigates the finite-time consensus problem of leader-following multiagent systems. The dynamical models for all following agents and the leader are assumed the same general form of linear system, and the interconnection topology among the agents is assumed to be switching and undirected. We mostly consider the continuous-time case. By assuming that the states of neighbouring agents are known to each agent, a sufficient condition is established for finite-time consensus via a neighbor-based state feedback protocol. While the states of neighbouring agents cannot be available and only the outputs of neighbouring agents can be accessed, the distributed observer-based consensus protocol is proposed for each following agent. A sufficient condition is provided in terms of linear matrix inequalities to design the observer-based consensus protocol, which makes the multiagent systems achieve finite-time consensus under switching topologies. Then, we discuss the counterparts for discrete-time case. Finally, we provide an illustrative example to show the effectiveness of the design approach.

## 1. Introduction

Cooperative control of multiagent systems (MAS) has received increasing attention over the last ten years with rather diverse background such as biology, physics, mathematics, information science, computer science, and control science. Many topics such as swarm, aggregation, formation, schooling, and synchronization are involved in a critical problem known as the consensus problem [1–5]. The objective of consensus for multiagent systems is to design the distributed protocols based on the local relative information so that the states of a team of agents can reach an agreement [1].

The consensus problems have a long history in the field of computer science. In [1], Jadbabaie et al. studied the consensus protocols motivated by biological group behaviors, which stirred the excitement of the research on distributed cooperative control in the control community. In most existing works on consensus, the agent dynamics are restricted to first-, second- and, sometimes, high-order integrators [1, 3, 6–12]. In [7], Ren and Atkins showed that in sharp contrast to the first-order consensus problem, consensus for a group of agents with second-order dynamics many fail to be achieved even if the network topology has a directed spanning tree. Recently, the consensus problem with a general linear dynamical agent has been probed by [13–19]. The interacting topology of multiagent systems is a key factor to achieve consensus. For fixed topology, the eigenvalue decomposition method can be used to solve the multiagent consensus problem [10, 14, 16]. For multiagent systems with high-order dynamics under switching interacting topology, the common Lyapunov function method is involved to analyze consensus problem of multiagent systems [6, 9, 17, 18].

Since some state variables cannot be obtained directly in many practical systems, the state observer is involved in proposed control law to achieve control aim. Till now, the observer-based design technique became an important control approach. Much of the attention has been devoted to achieving state consensus for a network of identical agents, where each agent has access to a linear combination of its own states relative to those of neighboring agents [1, 7, 10, 13]. In many practical systems, the agent cannot obtain full state information but only obtain output information of its neighbors. Usually, observer-based approach is proposed for agent to solve the state consensus problem. Distributed estimation via observers design for multiagent coordination is an important topic with wide applications especially in sensor networks and robot networks. To track the active leader, the tracking protocols based on state observers were proposed for the first-order and second-order agents [6, 9]. The observer-based protocols were provided to solve multiagent consensus problem with general linear dynamics in [14–19]. The leader-following configuration is very useful to design the multiagent systems, which has been discussed in [1, 6, 9, 13, 16–19].

Most of the existing control techniques related to the stability focus on Lyapunov asymptotic stability, which is defined over an infinite-time interval. However, in some practical applications, we mainly concern the behaviors of the system over a fixed finite-time interval, such as convergence to an equilibrium state in finite time. For these cases, the finite-time stability (FTS) is involved. Finite-time convergence to a Lyapunov stable equilibrium was investigated in [20]. Finite-time stabilization for a chain of power-integrator systems was considered in [21, 22]. A general framework for finite-time stability analysis based on vector Lyapunov functions was developed in [23]. The concept of FTS has been revisited by [24–28], which provided operative test conditions in light of linear matrix inequality (LMI). More recently, the concept of FTS was generalized to the finite-time consensus. In [29], Sun et al. studied the finite-time consensus problems of the leader-following multiagent systems with jointly reachable leader and switching jointly reachable leader. The finite-time synchronization between two complex networks with nondelayed and delayed was proposed by using the impulsive control and the periodically intermittent control in [30]. The consensus problems of second-order multiagent systems in the presence of one and multiple leaders under a direction graph were investigated in [31].

Motivated by the concept of finite-time stability (FTS) which was first introduced in the control literature by Dorato in [32] and correspondingly previous works (see [24–28]), we extend the concept of FTS to finite-time consensus (FTC), which is different from the concept involved in [29–31]. Compared with classical Lyapunov consensus problem, finite-time consensus here is an independent concept, which concerns the consensus of multiagent systems over a finite-time interval and may play an important part in the study of the transient behavior of system. First, we discuss the continuous-time FTC problem. Then, the discrete-time counterpart is probed. The dynamical model of agents is assumed as a general form of linear system, and the interconnection topology among the agents is assumed to be switching. While the full state information cannot be available, observer-based consensus protocols are provided to solve FTC problem. In light of LMI, we present some computationally appealing conditions to construct the gain matrices involved in the proposed protocols. Because the proposed consensus protocols are distributed, the computational complexity of design technique is only dependent on the dimension of agent's state and independent on the number of agents. LMI conditions can be solved effectively by interior-point method, and a number of software packages such as MATLAB LMI Toolbox can be available to solve LMI problems [33].

The subsequent sections are organized as follows. In Section 2, the formulation of finite-time consensus is given. Sufficient condition for finite-time consensus via state feedback and for existence of an output feedback controller guaranteeing finite-time consensus is provided, respectively, in Sections 3 and 4. This condition requires solution of an LMI problem. And discrete-time multiagent systems are investigated in Section 5. An illustrative example to verify the effectiveness of the theoretical results is provided in Section 6. Conclusion remarks are drawn in Section 7.

## 2. Preliminaries and Problem Formulation

### 2.1. Notations and Graph Theory

We first introduce the notations used in this paper. *R* (or *C*) is the real (or complex) number set. *I* denotes an appropriate dimensioned identity matrix and 1 denotes a column vector with all components equal to one. For a given matrix *A*, *A* ^*T*^ denotes its transpose and *A* ^−1^ denotes its inverse. *λ*_max⁡_(*A*) and *λ*_min⁡_(*A*) represent the maximum and minimum eigenvalues of matrix *A* with real spectrum, respectively. For a symmetric matrix *P*, by *P* > 0 (≥0, <0, or ≤0), we mean that *P* is positive definite (positive semidefinite, negative, or negative semidefinite). ||·|| denotes Euclidean norm. The condition number of matrix *A* is denoted by cond(*A*) = ||*A*||·||*A* ^−1^||. Furthermore, cond(*Q*) = *λ*_max⁡_(*Q*)/*λ*_min⁡_(*Q*) for positive definite *Q*. ⊗ denotes the Kronecker product, which satisfies the following: (1) (*A* ⊗ *B*)(*C* ⊗ *D*) = (*AC*)⊗(*BD*) and (2) if *A* ≥ 0 and *B* ≥ 0, then *A* ⊗ *B* ≥ 0.

We use an undirected graph to describe the involved information interaction topology, which is modeled by *G* = (*V*, *E*), where *V* = {*v*_1_, *v*_2_,…, *v*_*N*_} is the set of vertices representing *N* agents and *E*⊆*V* × *V* is the edges set. *v*_*j*_ is called a neighbor of *v*_*i*_ if (*v*_*i*_, *v*_*j*_) ∈ *E*, and the neighbor set of vertex *v*_*i*_ is denoted as *N*_*i*_ = {*j* | (*v*_*i*_, *v*_*j*_) ∈ *E*}. *W* = [*w*_*ij*_]_*N*×*N*_ represents weighted adjacency matrix associated with graph *G*, where *w*_*ij*_ > 0 if (*v*_*i*_, *v*_*j*_) ∈ *E* and *w*_*ij*_ = 0 otherwise. Correspondingly, the Laplacian matrix *L* is defined as *l*_*ii*_ = ∑_*j*=1,*j*≠*i*_^*N*^ *w*_*ij*_ and *l*_*ij*_ = −*w*_*ij*_.

We use $\overset{-}{G}$ of order *N* + 1 to model the interaction topology of the leader-following multiagent system, where the leader is represented by vertex *v*_0_. $\overset{-}{G}$ contains a subgraph *G* and *v*_0_ with the directed edges from some agents to the leader, where *G* described the interaction topology of *N* following agents. Note that the graph describing the interaction topology can vary with time. Suppose that the interconnection topology is switched among finite possible interconnection graphs, which is denoted as $S=\{{\overset{-}{G}}_{1},{\overset{-}{G}}_{2},\ldots ,{\overset{-}{G}}_{M_{0}}\}$ with index set *P* = {1,2,…, *M*_0_}. The switching signal *σ*(*t*) → *P* is used to express the index of topology graph. Certainly, it is assumed that the chatter does not occur; that is, *σ* switches finite times in any bounded time interval.

Next, we introduce following well-known result, which will be used in the sequel.

**Lemma 1 (see [34])**

Let *S* be a symmetric matrix with the partitioned form *S* = [*S*_*ij*_], where *S*_11_ ∈ *R* ^*r*×*r*^, *S*_12_ ∈ *R* ^*r*×(*n*−*r*)^, and *S*_22_ ∈ *R* ^(*n*−*r*)×(*n*−*r*)^. Then *S* < 0 if and only if

(1) $$\begin{array}{c} S_{11}<0,\;\;S_{22}-S_{21}S_{11}^{-1}S_{12}<0 \end{array}$$

or equivalently

(2) $$\begin{array}{c} S_{22}<0,\;\;S_{11}-S_{12}S_{22}^{-1}S_{21}<0. \end{array}$$

### 2.2. Problem Formulation

Consider an MAS consisting of *N* following agents and a leader agent. The dynamics of agent *i* is

(3) x˙i(t)=Axi(t)+Bui(t),yi(t)=Cxi(t),i=1,2,…,N,

where *x*_*i*_ ∈ *R* ^*n*^ is the agent *i*'s state, *u*_*i*_ ∈ *R* ^*m*^ is agent *i*'s control input, and *y*_*i*_ ∈ *R* ^*p*^ is the agent *i*'s measured output. *A*, *B*, *C* are constant matrices with appropriate dimensions. We always assume that the system satisfies following property.

**Assumption 2**

For system (3), (*A*, *B*) is stabilizable and (*A*, *C*) is observable.

The leader is an isolated agent and labeled as *v*_0_, which is described by

(4) x˙0(t)=Ax0(t)+Bu0(t),y0(t)=Cx0(t),

where *x*_0_ ∈ *R* ^*n*^ is the leader's state and *y*_0_ ∈ *R* ^*p*^ is the leader's measured output. The input *u*_0_(*t*) can be regarded as the common policy which is known by all following agents. Without loss of generality, we can assume that *u*_0_(*t*) = 0. The leader-following multiagent system modeled by (3) and (4) has been investigated in many references such as [13, 16–18].

The goal of this paper is to find some sufficient conditions which guarantee the existence of a dynamic feedback controller for leader-following multiagent systems such that the consensus can be achieved over the finite interval [0, *T*]. Let *x* = (*x*_1_^*T*^, *x*_2_^*T*^,…, *x*_*N*_^*T*^)^*T*^. Based on the tracking error *x* − 1*x*_0_, the concept of leader-following finite-time consensus can be formalized through the following definition, which is an extension to multiagent systems of the one give in [32].

**Definition 3 (leader-following finite-time consensus)**

Given three positive scalars *c*_1_, *c*_2_, *T*, with *c*_1_ < *c*_2_, and a positive definite matrix *R*, the system (3)-(4) is said to be FTC with respect to (*c*_1_, *c*_2_, *T*, *R*), if

(5) (x(0)−1x0(0))T(I⊗R)(x(0)−1x0(0)) ≤c1⟹(x(t)−1x0(t))T(I⊗R)(x(t)−1x0(t)) ≤c2, ∀t∈[0,T].

**Remark 4**

The linear system $\dot{x}(t)=Ax(t)$, *x*(0) = *x*_0_, is said to be FTS with respect to (*c*_1_, *c*_2_, *T*, *R*), if

(6) $$\begin{array}{c} x_{0}^{T}Rx_{0}\leq c_{1}⟹x^{T}\left(t\right)Rx\left(t\right)<c_{2},\;\forall t\in \left[0,T\right]. \end{array}$$

Lyapunov asymptotic stability and FTS are independent concepts: a system which is FTS may not be Lyapunov asymptotically stable; conversely a Lyapunov asymptotically stable system could not be FTS if, during the transients, its state exceeds the prescribed bounds [32].

## 3. Finite-Time Consensus with State Feedback

In this section, we investigate the finite-time consensus problem via distributed state feedback control protocol. The proposed protocol for the following agent *i*, which is based on the relative state error of agent *i* with its neighbor agents, is given as follows:

(7) ui(t)=−cK[∑j∈Ni(t)aij(t)(xi(t)−xj(t))    +di(t)(xi(t)−x0(t))],

where *c* is the positive coupling strength, *a*_*ij*_(*t*), (*i*, *j* = 1,2,…, *N*) and *d*_*i*_(*t*), (*i* = 1,2,…, *N*) are connection weights, which are chosen as follows:

(8) $$\begin{array}{c} a_{ij}\left(t\right)=\{\begin{array}{cc} \alpha_{ij} & \text{if}\;\text{agent}\;i\;\text{is}\;\text{connected}\;\text{to}\;\text{agent}\;j,\\ 0 & \text{otherwise}, \end{array}\\ d_{i}\left(t\right)=\{\begin{array}{cc} \beta_{i} & \text{if}\;\text{agent}\;i\;\text{is}\;\text{connected}\;\text{to}\;\text{the}\;\text{leader},\\ 0 & \text{otherwise}, \end{array} \end{array}$$

where *α*_*ij*_ is connection weight constant between agent *i* and agent *j*, and *β*_*i*_ > 0  (*i* = 1,…, *N*) is connection weight constant between agent *i* and leader.

Let *x*(*t*) = (*x*_1_^*T*^(*t*), *x*_2_^*T*^(*t*),…, *x*_*N*_^*T*^(*t*))^*T*^ ∈ *R* ^*Nn*^. Then, the overall system dynamics is

(9) x˙(t)=[IN⊗A−Lσ(t)⊗(cBK)]x(t)−[Dσ(t)⊗(cBK)](x(t)−1⊗x0(t)),

where *L*_*σ*(*t*)_ is the Laplacian matrix of the interaction graph *G*_*σ*(*t*)_ and *D*_*σ*(*t*)_ is an *N* × *N* diagonal matrix whose *i*th diagonal element is *d*_*i*_(*t*). For convenience, let *H*_*σ*(*t*)_ = *L*_*σ*(*t*)_ + *D*_*σ*(*t*)_.

**Lemma 5 (see [6])**

If graph $\overset{-}{G}$ is connected and undirected, then the symmetric matrix *H* is positive definite.

Since we assume that the graphs ${\overset{-}{G}}_{\sigma (t)}$ are always connected, then *H*_*σ*(*t*)_ are positive definite. According to Lemma 5 and the fact that *S* is a finite set, define $\overset{-}{\lambda }≔\min\{\lambda_{\min}(H_{p}):p\in S\}$, $\hat{\lambda }≔\max\{\lambda_{\max}(H_{p}):p\in S\}$, which are well defined and positive.

The parameter matrix *K* and the coupling gain *c* can be constructed as follows.

**Algorithm 6**

(1) Let *P* be a solution of the inequality

(10) $$\begin{array}{c} PA^{T}+AP-BB^{T}-\alpha P<0, \end{array}$$

where *α* is a nonnegative scalar. Choose the feedback gain matrix *K* as

(11) $$\begin{array}{c} K=\frac{1}{2}B^{T}P^{-1}. \end{array}$$

(2) Select the coupling gain *c* satisfied as

(12) $$\begin{array}{c} c\geq \frac{1}{\overset{-}{\lambda }}. \end{array}$$

**Remark 7**

If (*A*, *B*) is stabilizable and $\overset{-}{Q}$ is a symmetric positive definite matrix, then the following Riccati equation

(13) $$\begin{array}{c} A^{T}P+PA-PBB^{T}P+\overset{-}{Q}=0 \end{array}$$

has a unique positive definite matrix *P* [35]. Since (*A*, *B*) is stabilizable, we know that (*A* − (1/2)*αI*, *B*) is stabilizable too. Thus, for any positive definite $\overset{-}{Q}$, the following Riccati equation

(14) $$\begin{array}{c} {\left(A-\frac{1}{2}\alpha I\right)}^{T}\overset{-}{P}+\overset{-}{P}\left(A-\frac{1}{2}\alpha I\right)-\overset{-}{P}BB^{T}\overset{-}{P}+\overset{-}{Q}=0 \end{array}$$

has a unique positive definite matrix $\overset{-}{P}$. Let $P={\overset{-}{P}}^{-1}>0$, which satisfies (10). Therefore, the LMI (10) is solvable.

Now we can obtain the following result.

**Theorem 8**

Consider the multiagent system (3)-(4) whose topology graph ${\overset{-}{G}}_{\sigma (t)}$ that is associated with any interval [*t*_*j*_, *t*_*j*+1_) is undirected and connected. The feedback gain matrix *K* and the coupling strength *c* are able to be constructed by Algorithm 6. If the positive definite matrix *Q* = *R* ^1/2^ *P* ^−1^ *R* ^1/2^ satisfies the following condition:

(15) $$\begin{array}{c} \text{cond}\left(Q\right)<\frac{c_{2}}{c_{1}}e^{-\alpha T}, \end{array}$$

then under the state feedback controller (7), the leader-following multiagent system (3) and (4) is finite-time consensus with respect to (*c*_1_, *c*_2_, *T*, *R*).

**Proof**

Denote *ε*(*t*) = *x*(*t*) − 1 ⊗ *x*_0_(*t*), which represents the tracking error vector. In view of (4), (9) and *L*_*σ*(*t*)_1 = 0, the dynamics of tracking error is expressed as

(16) $$\begin{array}{c} \dot{\varepsilon }\left(t\right)=\left(I_{N}⊗A-cH_{\sigma (t)}⊗BK\right)\varepsilon \left(t\right). \end{array}$$

Then, the leader-following finite-time consensus problem is converted into finite-time stability problem.

Let *P* be a solution of (10) such that the condition (15) is satisfied. Consider the following common Lyapunov function:

(17) $$\begin{array}{c} V\left(t\right)=\varepsilon^{T}\left(t\right)\left(I_{N}⊗\overset{-}{P}\right)\varepsilon \left(t\right), \end{array}$$

where $\overset{-}{P}=P^{-1}$. Let *σ*(*t*) = *p*. The derivative of (17) along the trajectories of (16) yields

(18) V˙(t)=εT(t)[IN⊗(ATP−+P−A)−c(HpT+Hp)⊗P−BK]ε(t)=εT(t)[IN⊗(ATP−+P−A)−12c(HpT+Hp)⊗P−BBTP−]ε(t)≤εT(t)[IN⊗(ATP−+P−A−P−BBTP−)]ε(t)=εT(t)[IN⊗P−(PAT+AP−BBT)P−]ε(t)<εT(t)(IN⊗αP−)ε(t)=αV(t).

By integrating inequality (18) between 0 and *t* it follows that

(19) $$\begin{array}{c} V\left(\varepsilon \left(t\right)\right)<V\left(\varepsilon \left(0\right)\right)e^{\alpha t}. \end{array}$$

By the fact $\overset{-}{P}=R^{1/2}QR^{1/2}$, we can get the following chain of inequalities:

(20) V(ε(t))≥λmin⁡(Q)εT(t)(IN⊗R)ε(t),V(ε(0))eαt≤λmax⁡(Q)εT(0)(IN⊗R)ε(0)eαt≤λmax⁡(Q)c1eαt.

Putting together (19) and (20), we have

(21) $$\begin{array}{c} \varepsilon^{T}\left(t\right)\left(I_{N}⊗R\right)\varepsilon \left(t\right)<\frac{\lambda_{\max}\left(Q\right)}{\lambda_{\min}\left(Q\right)}c_{1}e^{\alpha t}<\frac{c_{2}}{c_{1}}e^{-\alpha T}c_{1}e^{\alpha t}<c_{2}. \end{array}$$

From (21), the proof is complete.

**Remark 9**

LMI (10) must be solvable for any *α* ≥ 0. Let Γ(*α*) be the positive definite solution set of (10) with parameter *α* ≥ 0. It is easy to see that while 0 ≤ *α*_1_ < *α*_2_, Γ(*α*_1_) ⊂ Γ(*α*_2_). Additionally, if *c*_2_/*c*_1_ is big enough, then condition (15) must hold. By (15), consider the optimization problem:

(22) $$\begin{array}{c} \underset{\alpha >0,P\in \Gamma (\alpha )}{\min}\text{cond}\left(R^{1/2}P^{-1}R^{1/2}\right)e^{\alpha T}. \end{array}$$

Obviously, we can construct *P* and *α* satisfying (15) based our design approach, while *c*_2_/*c*_1_ is greater than the optimal value of (22). Then, the finite-time consensus problem with respect to (*c*_1_, *c*_2_, *T*, *R*) can be solved by the proposed protocol in this case. Furthermore, if there exists *P* such that (10) and (15) with *α* = 0 are satisfied, it is not difficult to obtain $\dot{V}(x)<0$ from (18), which means that the leader-following multiagent system (3) and (4) is not only finite-time consensus but also asymptotically consensus. Obviously, condition (15) is satisfied if there exists a positive definite solution *P* of (10) such that the following LMI holds:

(23) $$\begin{array}{c} \lambda_{0}e^{\alpha T}R^{-1}<P<\lambda_{0}\frac{c_{2}}{c_{1}}R^{-1}, \end{array}$$

with positive constant *λ*_0_. Once a value for *α* is fixed, the design of a state feedback controller to make multiagent system achieve finite-time consensus is to solve LMIs (10) and (23). The LMI problems can be solved by a number of software packages such as the LMI Control Toolbox of MATLAB [33].

## 4. Finite-Time Consensus with State Observer

This section investigates the finite-time consensus problem with state observer-based protocol. In some practical systems, the full state is unavailable. At time *t*, agent *i* At time *t*, the relative output error with its neighbor agents can be available for agent *i*, which is denoted by, which is denoted by

(24) $$\begin{array}{c} \xi_{i}\left(t\right)=\underset{j\in N_{i}\left(t\right)}{\sum }a_{ij}\left(t\right)\left(y_{i}\left(t\right)-y_{j}\left(t\right)\right)+d_{i}\left(t\right)\left(y_{i}\left(t\right)-y_{0}\left(t\right)\right). \end{array}$$

To solve the leader-following multiagent finite-time consensus problem, consider the Luenberger observer for agent *i* with form

(25) v˙i(t)=Avi(t)+Bui(t)−cG[∑j∈Ni(t)aij(t)C(vi(t)−vj(t))+di(t)Cvi(t)−ξi(t)], vi(0)=0,

where *v*_*i*_ ∈ *R* ^*n*^ is the protocol state, *c* is the coupling strength, and *G* ∈ *R* ^*n*×*p*^ is a given gain matrix.

The feedback controller is

(26) $$\begin{array}{c} u_{i}\left(t\right)=-Kv_{i}\left(t\right), \end{array}$$

where *K* is a given feedback gain matrix. It is assumed that conditions (10) and (15) are solvable and *K* is designed by (11).

Let *ε*_*i*_(*t*) = *x*_*i*_(*t*) − *x*_0_(*t*), *e*_*i*_(*t*) = *ε*_*i*_(*t*) − *v*_*i*_(*t*), *ε*(*t*) = [*ε*_1_^*T*^(*t*),…, *ε*_*N*_^*T*^(*t*)]^*T*^, and *e*(*t*) = [*e*_1_^*T*^(*t*),…, *e*_*N*_^*T*^(*t*)]^*T*^. Then, we can get

(27) $$\begin{array}{c} \dot{\varepsilon }\left(t\right)=I_{N}⊗\left(A-BK\right)\varepsilon \left(t\right)+\left(I_{N}⊗BK\right)e\left(t\right), \end{array}$$

(28) $$\begin{array}{c} \dot{e}\left(t\right)=\left(I_{N}⊗A-cH_{\sigma (t)}⊗GC\right)e\left(t\right), \end{array}$$

with *ε*(0) = *x*(0) − 1 ⊗ *x*_0_(0) and *e*(0) = *x*(0) − 1 ⊗ *x*_0_(0).

Therefore the system state evolution is determined by the closed loop *I*_*N*_ ⊗ (*A* − *BK*) and by the behavior of the exogenous input *e*(*t*). The goal of this section is to design an observer gain *G* in (25) such that the leader-following FTC property of the system is not lost in the presence of the estimation error. If such a control gain *G* exists, the corresponding observer is also a dynamic output feedback controller which can solve the following problem. Certainly, the existence of such a controller implies finite-time consensus via state feedback. Therefore, without loss of generality, we present the following assumption.

**Assumption 10**

A state feedback matrix *K* which guarantees the leader-following multiagent finite-time consensus via state feedback exists and has been designed using the results of Theorem 8.

In the sequel, we try to solve the following observer-based finite-time consensus problem.

**Problem 11 (FTC via observer-based output feedback)**

Given a gain matrix *K* such that the multiagent system (3)-(4) is FTC wrt (*c*_1_, *c*_2_, *T*, *R*) via state feedback, find an observer gain *G* such that system (27) is FTC wrt (*c*_1_, *c*_2_, *W*_*G*_, *T*, *R*), where *W*_*G*_ is the set

(29) WG≔{e(t) ∣ e˙(t)=(IN⊗A−cHσ(t)⊗GC)e(t),  e(0)=ε(0),εT(0)(IN⊗R)ε(0)≤c1}.

From (27) and (28), the tracking error dynamical system can be expressed as

(30) $$\begin{array}{c} \dot{\eta }\left(t\right)=F_{\sigma (t)}\eta \left(t\right), \end{array}$$

where *η* = (*ε* ^*T*^, *e* ^*T*^)^*T*^ and

(31) $$\begin{array}{c} F_{\sigma (t)}=\left(\begin{array}{cc} I_{N}⊗\left(A-BK\right) & I_{N}⊗\left(BK\right)\\ 0 & I_{N}⊗A-cH_{\sigma (t)}⊗\left(GC\right) \end{array}\right). \end{array}$$

Obviously, the finite-time stability of system (30) implies that the finite-time consensus of leader-following system (3)-(4). Thus, the leader-following finite-time consensus problem of multiagent system is transformed into the finite-time stability problem of error dynamic system (30).

Now, we can present our main result as follows.

**Theorem 12**

Consider the multiagent system (3)-(4) whose topology graph ${\overset{-}{G}}_{\sigma (t)}$ that is associated with any interval [*t*_*j*_, *t*_*j*+1_) is undirected and connected.  Problem 11 is solvable if, letting $c\geq 1/\overset{-}{\lambda }$, *P*_1_ = *R* ^1/2^ *Q*_1_ *R* ^1/2^, and *P*_2_ = *R* ^1/2^ *Q*_2_ *R* ^1/2^, there exist a nonnegative scalar *α*, two symmetric positive definite matrices *Q*_1_ and *Q*_2_, and positive scalars *λ*_*k*_, *k* = 1,2, 3, such that

(32) $$\begin{array}{c} \left(\begin{array}{cc} A^{T}P_{1}+P_{1}A-P_{1}BK-K^{T}B^{T}P_{1}-\alpha P_{1} & P_{1}BK\\ {\left(P_{1}BK\right)}^{T} & A^{T}P_{2}+P_{2}A-C^{T}C-\alpha P_{2} \end{array}\right)<0, \end{array}$$

(33a) $$\begin{array}{c} \lambda_{3}I<Q_{1}<\lambda_{1}I, \end{array}$$

(33b) $$\begin{array}{c} 0<Q_{2}<\lambda_{2}I, \end{array}$$

(33c) $$\begin{array}{c} c_{1}\left(\lambda_{1}+\lambda_{2}\right)\leq c_{2}e^{-\alpha T}\lambda_{3}. \end{array}$$

In this case the consensus protocols (25) and (26) with gain matrix *G* = (1/2)*P*_2_^−1^ *C* ^*T*^ can make the multiagent system (3)-(4) FTC with respect to (*c*_1_, *c*_2_, *W*_*G*_, *T*, *R*).

**Proof**

Set *σ*(*t*) = *p*, *p* ∈ {1,2,…, *M*_0_}. Since *H*_*p*_ is symmetric, there exists an orthogonal matrix *T*_*p*_ such that

(34) $$\begin{array}{c} T_{p}H_{p}T_{p}^{T}=\Lambda_{p}=\mathrm{diag}\left(\lambda_{1}p,\lambda_{2}p,\ldots ,\lambda_{N}p\right), \end{array}$$

where *λ*_*i*_ *p* is the *i*th eigenvalue of *H*_*p*_. By using the following orthogonal transformation to system (30):

(35) $$\begin{array}{c} \tilde{\eta }=\left(\begin{array}{cc} I_{N}⊗I_{n} & 0\\ 0 & T_{p}⊗I_{n} \end{array}\right)\eta , \end{array}$$

we can get the equivalent system of system (30) as

(36) $$\begin{array}{c} \dot{\tilde{\eta }}\left(t\right)={\tilde{F}}_{p}\tilde{\eta }\left(t\right), \end{array}$$

where $\tilde{\eta }={\left({\tilde{\varepsilon }}^{T},{\tilde{e}}^{T}\right)}^{T}$ and

(37) $$\begin{array}{c} {\tilde{F}}_{p}=\left(\begin{array}{cc} I_{N}⊗\left(A-BK\right) & I_{N}⊗BK\\ 0 & I_{N}⊗A-c\Lambda_{p}⊗GC \end{array}\right). \end{array}$$

That is

(38) $$\begin{array}{c} {\dot{\tilde{\eta }}}_{i}\left(t\right)=\left(\begin{array}{cc} A-BK & BK\\ 0 & A-c\lambda_{ip}GC \end{array}\right){\tilde{\eta }}_{i}\left(t\right), \end{array}$$

where ${\tilde{\eta }}_{i}={\left({\tilde{\varepsilon }}_{i}^{T},{\tilde{e}}_{i}^{T}\right)}^{T}$.

Consider the following Lyapunov function:

(39) $$\begin{array}{c} V\left(\eta \left(t\right)\right)=\eta^{T}\left(t\right)\tilde{P}\eta \left(t\right), \end{array}$$

where

(40) $$\begin{array}{c} \tilde{P}=\left(\begin{array}{cc} I_{N}⊗P_{1} & 0\\ 0 & I_{N}⊗P_{2} \end{array}\right), \end{array}$$

*V*(*η*(*t*)) is continuously differentiable at any time except for switching instants.

Consider

(41) [IN⊗(A−BK)]T(IN⊗P1)  +(IN⊗P1)[IN⊗(A−BK)] =IN⊗(ATP1+P1A−P1BK−KTBTP1).

Noting that *P*_2_ *GC* = (1/2)*C* ^*T*^ *C*, we have

(42) (IN⊗A−cHp⊗(GC))T(IN⊗P2)  +(I⊗P2)(I⊗A−cHp⊗(GC)) =IN⊗(ATP2+P2A)−12c(HpT+Hp)⊗CTC <IN⊗(ATP2+P2A−CTC).

Then derivative of (39) along the trajectories of (30) yields

(43) V˙(η(t))=ηT(t)(FpTP~+P~Fp)η(t)=η~T(t)(F~pTP~+P~F~p)η~(t)≤η~T(t)Qpη~(t)=∑i=1N η~iT(t)Qipη~i(t),‍

where

(44) Qip=(ATP1+P1A−P1BK−KTBTP1P1BK(P1BK)TATP2+P2A−CTC).

From (32), we obtain

(45) $$\begin{array}{c} Q_{ip}<\left(\begin{array}{cc} \alpha P_{1} & 0\\ 0 & \alpha P_{2} \end{array}\right), \end{array}$$

(46) $$\begin{array}{c} \dot{V}\left(t\right)<\alpha V\left(t\right). \end{array}$$

By integrating inequality (46) between 0 and *t* it follows that

(47) $$\begin{array}{c} V\left(\eta \left(t\right)\right)<V\left(\eta \left(0\right)\right)e^{\alpha t}. \end{array}$$

We have the following chain of inequalities:

(48) V(η(t))≥λmin⁡(Q1)εT(t)(IN⊗R)ε(t)+λmin⁡(Q2)eT(t)(IN⊗R)e(t)≥λmin⁡(Q1)εT(t)(IN⊗R)ε(t).

In additionally, one has

(49) V(η(0))eαt≤(λmax⁡(Q1)εT(0)(IN⊗R)ε(0) +λmax⁡(Q2)eT(0)(IN⊗R)e(0))eαt≤(λmax⁡(Q1)+λmax⁡(Q2))c1eαT.

Putting together (47), (48), and (49), we have

(50) $$\begin{array}{c} \varepsilon^{T}\left(t\right)\left(I_{N}⊗R\right)\varepsilon \left(t\right)<\frac{\left(\lambda_{\max}\left(Q_{1}\right)+\lambda_{\max}\left(Q_{2}\right)\right)}{\lambda_{\min}\left(Q_{1}\right)}c_{1}e^{\alpha T}. \end{array}$$

Since

(51) $$\begin{array}{c} \lambda_{3}<\lambda_{\min}\left(Q_{1}\right),\;\;\lambda_{\max}\left(Q_{1}\right)<\lambda_{1},\\ 0<\lambda_{\min}\left(Q_{2}\right),\;\;\lambda_{\max}\left(Q_{2}\right)<\lambda_{2},\\ \lambda_{1}c_{1}+\lambda_{2}c_{1}\leq c_{2}e^{-\alpha T}\lambda_{3}, \end{array}$$

which in turn guarantees that

(52) $$\begin{array}{c} \left(\lambda_{\max}\left(Q_{1}\right)+\lambda_{\max}\left(Q_{2}\right)\right)c_{1}<c_{2}e^{-\alpha T}\lambda_{\min}\left(Q_{1}\right), \end{array}$$

then we can get

(53) $$\begin{array}{c} \varepsilon^{T}\left(I_{N}⊗R\right)\varepsilon <\frac{1}{\lambda_{\min}\left(Q_{1}\right)}c_{2}e^{-\alpha T}\lambda_{\min}\left(Q_{1}\right)e^{\alpha T}=c_{2}, \end{array}$$

for all *t* ∈ [0, *T*].

## 5. Discrete-Time Multiagent Systems

This section focuses on the discrete-time counterpart of the last section. Consider a network of *N* identical discrete-time linear agents and one leader, with the dynamics of the *i*th agent described by

(54) xi(k+1)=Axi(k)+Bui(k), yi(k)=Cxi(k),i=1,2,…,N

and the dynamics of the leader is described by

(55) x0(k+1)=Ax0(k),y0(k)=Cx0(k).

**Definition 13 (leader-following finite-time consensus)**

Given three positive scalars *c*_1_, *c*_2_, *M* with *c*_1_ < *c*_2_, and a positive definite matrix *R*, the discrete-time multiagent system (54)-(55) is said to be finite-time consensus with respect to (*c*_1_, *c*_2_, *M*, *R*), if

(56) (x(0)−1⊗x0(0))T(IN⊗R)(x(0)−1⊗x0(0))≤c1 ⟹(x(k)−1⊗x0(k))T   ×(IN⊗R)(x(k)−1⊗x0(k))≤c2,k=1,2,…,M.

### 5.1. Discrete-Time Finite-Time Consensus with State Feedback

First, we investigate finite-time consensus problem via distributed state feedback control protocol. The proposed protocol for the following agent *i* is constructed as follows:

(57) ui(k)=− cK∑j∈Ni(k)aij(k)(xi(k)−xj(k))+di(k)(xi(k)−x0(k)).

Let *ε*(*k*) = *x*(*k*) − 1 ⊗ *x*_0_(*k*). Similarly, we can get

(58) $$\begin{array}{c} \varepsilon \left(k+1\right)=\left(I_{N}⊗A-cH_{\sigma (k)}⊗BK\right)\varepsilon \left(k\right). \end{array}$$

Here we present our result about discrete-time finite-time consensus with state feedback.

**Theorem 14**

Consider the multiagent system (54)-(55) whose topology graph ${\overset{-}{G}}_{\sigma (k)}$ that is associated with any interval [*t*_*j*_, *t*_*j*+1_) is undirected and connected. If there exists a positive definite matrix *Q* ∈ *R* ^*n*×*n*^, a matrix *S* ∈ *R* ^*m*×*n*^ and a scalar *γ* ≥ 1 such that

(59) $$\begin{array}{c} \left(\begin{array}{cc} -\gamma Q & {\left(AQ-c\overset{-}{\lambda }BS\right)}^{T}\\ \left(AQ-c\overset{-}{\lambda }BS\right) & -Q \end{array}\right)<0, \end{array}$$

(60) $$\begin{array}{c} \left(\begin{array}{cc} -\gamma Q & {\left(AQ-c\hat{\lambda }BS\right)}^{T}\\ \left(AQ-c\hat{\lambda }BS\right) & -Q \end{array}\right)<0, \end{array}$$

(61) $$\begin{array}{c} \frac{\lambda_{\max}\left(\tilde{Q}\right)}{\lambda_{\min}\left(\tilde{Q}\right)}<\frac{1}{\gamma^{M}}\frac{c_{2}}{c_{1}}, \end{array}$$

where $\tilde{Q}=R^{1/2}QR^{1/2}$, and the feedback gain matrix *K* is taken by *K* = *SQ* ^−1^. Then the multiagent system (54)-(55) is finite-time consensus with respect to (*c*_1_, *c*_2_, *R*, *M*).

**Proof**

Let *σ*(*k*) = *p*, *p* ∈ {1,2,…, *M*_0_}. Due to $\lambda_{ip}\in [\overset{-}{\lambda },\hat{\lambda }]$, there exist *α*_*ip*_ ≥ 0 and *β*_*ip*_ ≥ 0 satisfying $\lambda_{ip}=\alpha_{ip}\overset{-}{\lambda }+\beta_{ip}\hat{\lambda }$ and *α*_*ip*_ + *β*_*ip*_ = 1.

From (59), (60), we get

(62) (−γQ(AQ−cλipBS)T(AQ−cλipBS)−Q)  =αip(−γQ(AQ−cλ−BS)T(AQ−cλ−BS)−Q)   +βip(−γQ(AQ−cλ^BS)T(AQ−cλ^BS)−Q)<0.

Pre- and postmultiplying (62) by the symmetric matrix $\left(\begin{array}{cc} Q^{-1} & 0\\ 0 & I \end{array}\right)$, the following equivalent condition is obtained:

(63) $$\begin{array}{c} \left(\begin{array}{cc} -\gamma Q^{-1} & {\left(A-c\lambda_{ip}BK\right)}^{T}\\ \left(A-c\lambda_{ip}BK\right) & -Q \end{array}\right)<0. \end{array}$$

Consider the following common Lyapunov function:

(64) $$\begin{array}{c} V\left(\varepsilon \left(k\right)\right)=\varepsilon^{T}\left(k\right)\left(I_{N}⊗P\right)\varepsilon \left(k\right), \end{array}$$

where *P* = *Q* ^−1^; then we get

(65) V(ε(k+1))=εT(k+1)(IN⊗P)ε(k+1)=∑i=1N εiT(k)(A−cλipBK)T×P(A−cλipBK)εi(k),

where equation (63) implies

(66) $$\begin{array}{c} V\left(\varepsilon \left(k+1\right)\right)<\gamma V\left(\varepsilon \left(k\right)\right). \end{array}$$

Applying iteratively (66), we obtain

(67) $$\begin{array}{c} V\left(\varepsilon \left(k\right)\right)<\gamma^{k}V\left(\varepsilon \left(0\right)\right),\;k=1,2,\ldots ,M. \end{array}$$

Now letting $\tilde{P}=R^{-1/2}PR^{-1/2}$, it is obvious to see that $\lambda_{\min}(\tilde{P})=1/\lambda_{\max}(\tilde{Q})$ and $\lambda_{\max}(\tilde{P})=1/\lambda_{\min}(\tilde{Q})$. And using the fact that *γ* ≥ 1, we have

(68) γkV(ε(0))=γk[εT(0)(IN⊗P)ε(0)]≤γk[λmax⁡(P~)εT(0)(IN⊗R)ε(0)]≤γMλmax⁡(P~)c1,V(ε(k))=[εT(k)(IN⊗P)ε(k)]≥λmin⁡(P~)εT(k)(IN⊗R)ε(k).

Putting together (67) and (68), we obtain

(69) $$\begin{array}{c} \varepsilon^{T}\left(k\right)\left(I_{N}⊗R\right)\varepsilon \left(k\right)<\frac{\lambda_{\max}\left(\tilde{P}\right)}{\lambda_{\min}\left(\tilde{P}\right)}\gamma^{M}c_{1}=\frac{\lambda_{\max}\left(\tilde{Q}\right)}{\lambda_{\min}\left(\tilde{Q}\right)}\gamma^{M}c_{1}<c_{2}. \end{array}$$

Then, system (54)-(55) is finite-time consensus with respect to (*c*_1_, *c*_2_, *R*, *M*).

**Remark 15**

Once we have fixed a value for *γ*, the feasibility of the conditions stated in the Theorem 14 can be turned into LMI-based feasibility problem. To this aim, it is easy to check that condition (61) is guaranteed by imposing the conditions:

(70) $$\begin{array}{c} \mu_{1}R^{-1}<Q<R^{-1}, \end{array}$$

(71) $$\begin{array}{c} \frac{c_{1}}{\mu_{1}}<\frac{c_{2}}{\gamma^{M}}, \end{array}$$

for positive number *μ*_1_. Letting $\delta =\sqrt{c_{1}}$, inequality (71) is equivalent to the following LMI by using Schur Complement Lemma:

(72) $$\begin{array}{c} \left(\begin{array}{cc} \frac{c_{2}}{r^{M}} & \delta \\ \delta & \mu_{1} \end{array}\right)>0. \end{array}$$

### 5.2. Discrete-Time Finite-Time Consensus with State Observer

Now we consider that the full state of agent *i* is unknown, and we only know the output of agent *i*. At time *k*, agent *i* can be available to the relative output error with its neighbor agents, which is denoted by

(73) ξi(k)=∑j∈Ni(k)‍aij(k)(yi(k)−yj(k))+di(k)(yi(k)−y0(k)).

To solve the leader-following multiagent finite-time consensus problem, consider the discrete-time Luenberger observer for agent *i* with form

(74) vi(k+1)=Avi(k)+Bui(k)−cG[∑j∈Ni(k)aij(k)C(vi(k)−vj(k))+di(k)Cvi(k)−ξi(k)], vi(0)=0,

where *v*_*i*_(*k*) ∈ *R* ^*n*^ is the protocol state, *c* is the coupling strength, *G* ∈ *R* ^*n*×*p*^ is a gain matrix. The feedback controller is taken by

(75) $$\begin{array}{c} u_{i}\left(k\right)=-Kv_{i}\left(k\right), \end{array}$$

where *K* is a given feedback gain matrix.

Taking the similar step as the continuous-time case, we can get

(76) $$\begin{array}{c} \varepsilon \left(k+1\right)=\left[I_{N}⊗\left(A-BK\right)\right]\varepsilon \left(k\right)+\left(I_{N}⊗BK\right)e\left(k\right), \end{array}$$

with

(77) $$\begin{array}{c} e\left(k+1\right)=\left(I_{N}⊗A-cH_{\sigma (k)}⊗GC\right)e\left(k\right). \end{array}$$

The goal of this section is to design an observer gain *G* in (77) such that the leader-following FTC property of the system is not lost under given controller *K* in present of the estimation error. Here, we also assume that *K* has been designed by result of Theorem 14. Similarly, we consider the following problem.

**Problem 16 (FTC via observer-based output feedback)**

Given a gain matrix *K* such that the multiagent system (54)-(55) is FTC with respect to (*c*_1_, *c*_2_, *M*, *R*) via state feedback, find an observer gain *G* such that system (76) is FTC with respect to (*c*_1_, *c*_2_, *W*_*G*_, *M*, *R*), where *W*_*G*_ is the set

(78) WG≔{e(k) ∣ e(k+1)=(IN⊗A−cHσ(k)⊗GC)e(k), e(0)=ε(0),εT(0)(IN⊗R)ε(0)≤c1}.

Here we give our result for the discrete-time multiagent systems.

**Theorem 17**

Consider the multiagent system (54) and (55) whose topology graph ${\overset{-}{G}}_{\sigma (k)}$ that is associated with any interval [*t*_*j*_, *t*_*j*+1_) is undirected and connected. The problem is solvable if there exist positive-definite matrices *Q*_1_, *Q*_2_, a matrix *T*, and a scalar *γ* ≥ 1 such that

(79) $$\begin{array}{c} \left(\begin{array}{ccc} {\left(A-BK\right)}^{T}Q_{1}\left(A-BK\right)-\gamma Q_{1} & {\left(A-BK\right)}^{T}Q_{1}\left(BK\right) & 0\\ {\left(BK\right)}^{T}Q_{1}\left(A-BK\right) & {\left(BK\right)}^{T}Q_{1}\left(BK\right)-\gamma Q_{2} & {\left(Q_{2}A-c\overset{-}{\lambda }TC\right)}^{T}\\ 0 & \left(Q_{2}A-c\overset{-}{\lambda }TC\right) & -Q_{2} \end{array}\right)<0, \end{array}$$

(80) $$\begin{array}{c} \left(\begin{array}{ccc} {\left(A-BK\right)}^{T}Q_{1}\left(A-BK\right)-\gamma Q_{1} & {\left(A-BK\right)}^{T}Q_{1}\left(BK\right) & 0\\ {\left(BK\right)}^{T}Q_{1}\left(A-BK\right) & {\left(BK\right)}^{T}Q_{1}\left(BK\right)-\gamma Q_{2} & {\left(Q_{2}A-c\hat{\lambda }TC\right)}^{T}\\ 0 & \left(Q_{2}A-c\hat{\lambda }TC\right) & -Q_{2} \end{array}\right)<0, \end{array}$$

(81) $$\begin{array}{c} \frac{\lambda_{\max}\left({\tilde{Q}}_{1}\right)+\lambda_{\max}\left({\tilde{Q}}_{2}\right)}{\lambda_{\min}\left({\tilde{Q}}_{1}\right)}<\frac{1}{\gamma^{M}}\frac{c_{2}}{c_{1}}, \end{array}$$

where ${\tilde{Q}}_{1}=R^{-1/2}Q_{1}R^{-1/2}$ and ${\tilde{Q}}_{2}=R^{-1/2}Q_{2}R^{-1/2}$. In this case the consensus protocols (74) and (75) with gain matrix *G* = *Q*_2_^−1^ *T* can make the multiagent system (54)-(55) FTC with respect to (*c*_1_, *c*_2_, *W*_*G*_, *M*, *R*).

**Proof**

Let *σ*(*k*) = *p*, *p* ∈ {1,2,…, *M*_0_}. From (79) and (80), we get

(82) $$\begin{array}{c} \left(\begin{array}{ccc} {\left(A-BK\right)}^{T}Q_{1}\left(A-BK\right)-\gamma Q_{1} & {\left(A-BK\right)}^{T}Q_{1}\left(BK\right) & 0\\ {\left(BK\right)}^{T}Q_{1}\left(A-BK\right) & {\left(BK\right)}^{T}Q_{1}\left(BK\right)-\gamma Q_{2} & {\left(Q_{2}A-c\lambda_{ip}TC\right)}^{T}\\ 0 & \left(Q_{2}A-c\lambda_{ip}TC\right) & -Q_{2} \end{array}\right)<0. \end{array}$$

Obviously, (82) is equivalent to the following inequality:

(83) $$\begin{array}{c} \left(\begin{array}{cc} {\left(A-BK\right)}^{T}Q_{1}\left(A-BK\right)-\gamma Q_{1} & {\left(A-BK\right)}^{T}Q_{1}\left(BK\right)\\ {\left(BK\right)}^{T}Q_{1}\left(A-BK\right) & {\left(BK\right)}^{T}Q_{1}\left(BK\right)+{\left(A-c\lambda_{ip}GC\right)}^{T}Q_{2}\left(A-c\lambda_{ip}GC\right)-\gamma Q_{2} \end{array}\right)<0. \end{array}$$

Consider the following common Lyapunov function:

(84) V(ε(k),e(k))=εT(k)(IN⊗Q1)ε(k)+eT(k)(IN⊗Q2)e(k),

where equation (83) implies

(85) $$\begin{array}{c} V\left(\varepsilon \left(k+1\right),e\left(k+1\right)\right)<\gamma V\left(\varepsilon \left(k\right),e\left(k\right)\right). \end{array}$$

Applying iteratively (85), we obtain

(86) $$\begin{array}{c} V\left(\varepsilon \left(k\right),e\left(k\right)\right)<\gamma^{k}V\left(\varepsilon \left(0\right),e\left(0\right)\right),\;k=1,2,\ldots ,M. \end{array}$$

Since ${\tilde{Q}}_{1}=R^{-1/2}Q_{1}R^{-1/2}$, ${\tilde{Q}}_{2}=R^{-1/2}Q_{2}R^{-1/2}$ and the fact that *γ* ≥ 1, we have

(87) γkV(ε(0),e(0))=γk[εT(0)(IN⊗Q1)ε(0)+eT(0)(IN⊗Q2)e(0)]≤γk[λmax⁡(Q~1)εT(0)(IN⊗R)ε(0)+λmax⁡(Q~2)eT(0)(IN⊗R)e(0)]≤γk(λmax⁡(Q~1)+λmax⁡(Q~2))c1,

(88) V(ε(k),e(k))=εT(k)(IN⊗Q1)ε(k) +eT(k)(IN⊗Q2)e(k)≥λmin⁡(Q~1)εT(k)(IN⊗R)ε(k).

Putting together (86)–(88) we obtain

(89) $$\begin{array}{c} \varepsilon^{T}\left(k\right)\left(I_{N}⊗R\right)\varepsilon \left(k\right)\leq \frac{\lambda_{\max}\left({\tilde{Q}}_{1}\right)+\lambda_{\max}\left({\tilde{Q}}_{2}\right)}{\lambda_{\min}\left({\tilde{Q}}_{1}\right)}\gamma^{k}c_{1}<c_{2}. \end{array}$$

So the multiagent system (54)-(55) is finite-time consensus with respect to (*c*_1_, *c*_2_, *M*, *R*).

**Remark 18**

Once we have fixed a value for *γ*, the feasibility of the conditions stated in Theorem 17 can be turned into LMI-based feasibility problem. To this aim, it is easy to check that condition (81) is guaranteed by imposing the conditions

(90a) $$\begin{array}{c} \mu_{1}R<Q_{1}<R, \end{array}$$

(90b) $$\begin{array}{c} 0<Q_{2}<\mu_{2}R, \end{array}$$

(91) $$\begin{array}{c} \frac{1+\mu_{2}}{\mu_{1}}<\frac{c_{2}}{\gamma^{M}c_{1}}, \end{array}$$

for positive numbers *μ*_1_ and *μ*_2_. Similar, letting $\delta =\sqrt{1+\mu_{2}}$, inequality (91) is equivalent to the following LMI:

(92) $$\begin{array}{c} \left(\begin{array}{cc} \frac{c_{2}}{c_{1}\gamma^{M}} & \delta \\ \delta & \mu_{1} \end{array}\right)>0. \end{array}$$

## 6. Simulation Example

In this section, we discuss the numerical implementation of Theorem 8 with static feedback and Theorem 12 regarding the output feedback design. The group of agents consists of four following agents and one leader; that is *N* = 4. The leader agent and following agents are modeled by the linear dynamics (4) and (3), respectively, with the following system matrices:

(93) $$\begin{array}{c} A=\left(\begin{array}{ccc} -0.7 & -0.49 & 0.3\\ 1 & 0 & 0.4\\ 0.5 & 0 & -1.19 \end{array}\right),\;\;B=\left(\begin{array}{cc} 1 & 0\\ 0 & 0\\ 0 & 1 \end{array}\right),\\ C=\left(\begin{array}{ccc} 1 & 0.49 & 1.19\\ 0 & 0.49 & 1 \end{array}\right). \end{array}$$

**Figure 1:**
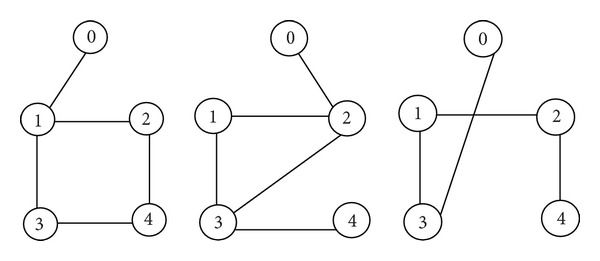
Three interconnection topology graphs.

The interconnection topologies are assumed to be arbitrarily switched among three graphs ${\overset{-}{G}}_{i}\;\;(i=1,2,3)$, which is shown in Figure 1.

The Laplacian matrices *L*_*i*_  (*i* = 1,2, 3) for subgraphs *G*_*i*_ are

(94) L1=(2−1−10−120−1−102−10−1−12),L2=(2−1−10−12−10−1−13−100−11),L3=(2−1−10−120−1−10100−101).

The diagonal matrices for the relationship between the leader and the followers are

(95) D1=diag⁡(1000),D2=diag⁡(0100),D3=diag⁡(0010).

By simple calculations, we can obtain $\hat{\lambda }=4.3429$ and $\overset{-}{\lambda }=0.1206$.

Take *c*_1_ = 1, *c*_2_ = 3, and *R* = *I*. Our goal is to find a dynamical feedback controller by which the multiagent system (3)-(4) is FTC with respect to (*c*_1_, *c*_2_, *T*, *R*).

(1) Choosing *α* = 0 and *T* = 3, with the aid of the LMI Control Toolbox, we can obtain control gain matrix
  (96) $$\begin{array}{c} K=\left(\begin{array}{ccc} 3.9835 & 5.2242 & 1.0326\\ 1.0326 & 1.9331 & 1.4431 \end{array}\right) \end{array}$$

and the gain matrix

(97) $$\begin{array}{c} G=\left(\begin{array}{cc} 0.0008 & -0.0002\\ -0.0002 & 0.0010\\ 0.0004 & 0.0000 \end{array}\right). \end{array}$$

**Figure 2:**
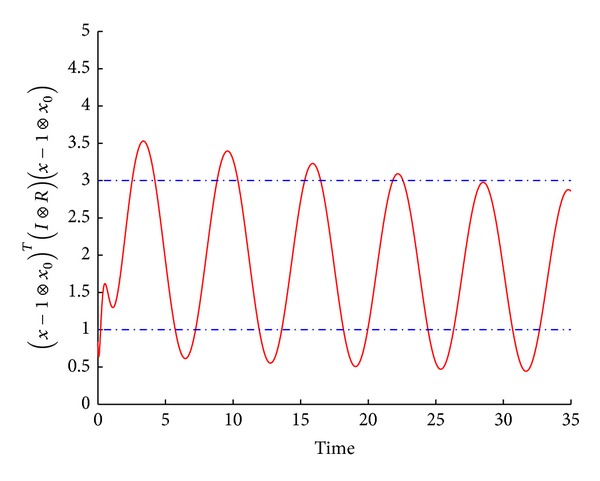
The error trajectories between the leader and each agent.

Figure 2 shows that the leader-following multiagent system is asymptotic consensus, but not finite-time consensus with respect to (*c*_1_, *c*_2_, *T*, *R*).

**Figure 3:**
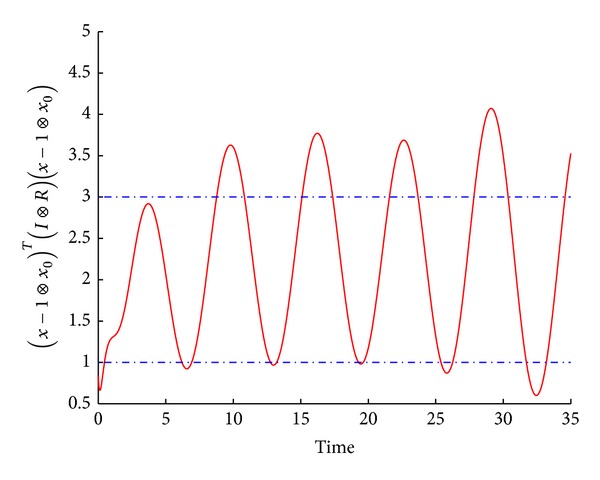
The error trajectories between the leader and each agent.

(2) Take *α* = 0.3 and *T* = 3. With the control gain matrix $K=\left(\begin{array}{ccc} 2.5797 & 1.7563 & -0.2964\\ -0.2964 & 0.1658 & 1.6599 \end{array}\right)$ and the gain matrix $G=\left(\begin{array}{cc} 0.0018 & -0.0014\\ -0.0005 & 0.0031\\ 0.0025 & 0.0018 \end{array}\right)$, the trajectories of tracking errors are depicted in Figure 3, which show that the multiagent system is finite-time consensus but not asymptotic consensus.
(3) Choose *α* = 0 and with the control gain matrix
  (98) $$\begin{array}{c} K=\left(\begin{array}{ccc} 0.3341 & 0.1807 & 0.0365\\ 0.0365 & 0.0591 & 0.2353 \end{array}\right) \end{array}$$

and the gain matrix

(99) $$\begin{array}{c} G=\left(\begin{array}{ccc} 0.2652 & -0.2133 & \\ -0.0446 & 0.5563 & \\ 0.4584 & 0.3170 & \end{array}\right). \end{array}$$

**Figure 4:**
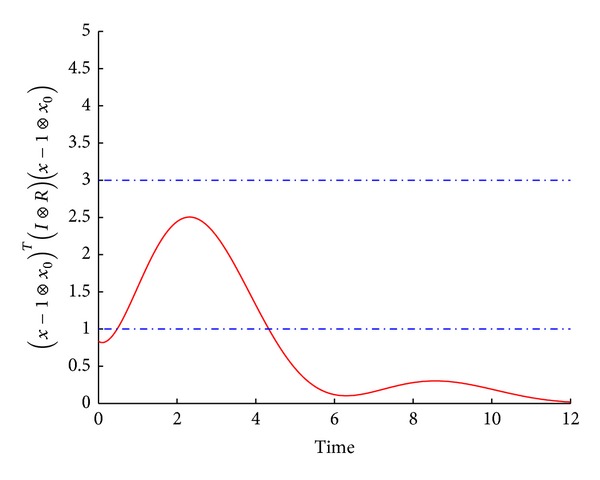
The error trajectories between the leader and each agent.

Figure 4 shows that the leader-following multiagent system is not only finite-time consensus with respect to (*c*_1_, *c*_2_, *T*, *R*) but also asymptotic consensus. From the above example, we know that when *a* = 0, the multiagent system can achieve asymptotic consensus, but maybe not finite-time consensus. Only when conditions (10) and (15) are both satisfied for *a* = 0, the multiagent system is both asymptotic consensus and finite-time consensus.

## 7. Conclusion

In this paper, we have discussed the finite-time consensus problem for leader-following multiagent systems with variable topology. Motivated by the concept of finite-time stability, the concept of finite-time consensus is proposed. The graph-theoretic notion is used to represent dynamical undirected interaction topologies. Two distributed consensus protocols based on its state and its output, respectively, are proposed to solve finite-time consensus. In light of linear matrix inequalities, some sufficient conditions are established to ensure that the multiagent system achieves finite-time consensus. Furthermore, we discuss the discrete-time counterpart along the similar lines. The simulation example also shows the effectiveness of the obtained theoretical results. There are some other observer/controller architectures that have been proposed to solve multiagent consensus problem. Our proposed design method can be also applied to solve the finite-time consensus problem under those architectures. Future extensions will focus on switching directed interaction topology, disturbance rejection, and robustness properties of the proposed consensus protocols.
